# I belong but I’m still sad: Reminders of Facebook increase feelings of belonging but do not facilitate coping with sadness

**DOI:** 10.1371/journal.pone.0209889

**Published:** 2018-12-31

**Authors:** Judith Knausenberger, Gerald Echterhoff

**Affiliations:** Department of Psychology, University of Münster, Münster, Germany; University Hospital of Tubingen, GERMANY

## Abstract

One way in which people may cope with sadness is to seek positive social contact. We examined whether subtle reminders of Facebook increase positive mood and thus attenuate the interest in social activities that is typically enhanced by sad mood induction. Participants watched either a loss-related sad or neutral video and were afterwards presented with either a Facebook, positive (sun) or neutral (Word) icon. We then examined their mood and their desire to engage in social activities as well as their feeling of belonging. The presentation of the Facebook icon increased feelings of belonging, but it did not influence participants’ other responses to the sad video. Participants reported more negative mood and a greater desire to engage in social activities after the sad (vs. control) video regardless of the icon condition. The results suggest that the activation of thoughts about Facebook can enhance users’ feeling of belonging; however, this effect might not be sufficient to facilitate coping with loss-related sadness.

## Introduction

When faced with sadness a typical reaction is to seek social support [1]. Nowadays, this social support can not only be found in real life but also online, in Social Networking Sites (SNS; [2]). The largest SNS is Facebook with more than a billion active users [3]. One of the main reasons to use Facebook is socializing with others [4], and people with higher belongingness needs use Facebook more frequently [5]. Thus, belongingness needs threatened by negative social experiences can be replenished by active Facebook use.

Interestingly, simply reminding people of Facebook via the subtle presentation of a Facebook icon can reduce threats posed by a prior experience of social exclusion (specifically, ostracism) [6]. As a consequence, socially excluded study participants seek opportunities for positive social contact and re-affiliation to a lower extent after subtle exposure to a Facebook icon (vs. a control icon such as the Flashplayer or Word icon). In other words, the activation of thoughts about Facebook can apparently help victims of exclusion to ward off threat to belonging. This, in turn, renders attempts for social reinclusion unnecessary.

Seeking social support is also a typical coping response to sadness [1]. Extending existing research, we examined whether reminding people of Facebook can serve as a source of social support and help people cope with sad mood. While sad people often withdraw from others, they are still likely to overcome sadness by seeking social contact [7]. Indeed, researchers have proposed that sadness may motivate people to engage in behavior that enhances the chance to maintain positive relationships [8]. This assumption is supported by empirical evidence that inducing sadness leads to a greater desire to engage in social activities [9]. This is, however, only the case for loss-related sadness, that is thinking about the loss of a loved one. In contrast, failure-related sadness does not increase the desire to engage in social activities ([9], Experiment 3).

Thus, when sadness is rooted in the fear of losing social connections interest in engaging in social activities is enhanced. Additionally, the experience of sadness is related to feelings of loneliness [10]. If, however, people experience socio-affective support, the effect of negative emotions on loneliness is reduced [11].Taken together, loss-related sadness should increase belongingness needs, which can be fulfilled by the experience of social support.

As an alternative to seeking direct personal support, people can restore belongingness needs by using social media sites such as Facebook [5]. A main reason to use Facebook is to stay in touch with social connections [4, 12], and Facebook use can help people cope with loneliness [13]. For example, active posting on Facebook reduces loneliness [14]. However, active Facebook use is not the only means for restoring a sense of belonging. Simple reminders of Facebook, specifically the presentation of a Facebook icon on a computer screen, can reduce threats to belongingness needs from social exclusion [6]. Also, reminding participants of Facebook reduces their interest in social contact that is typically induced by social exclusion [6, 15]. Thus, reminding people of Facebook when they experience loss-related sadness might fulfill their belongingness needs and thus help them to cope with sadness. This process should make other attempts to restore belongingness needs redundant.

Belongingness needs are not only influenced by the situation but can also vary depending on participants’ personality. One aspect that is associated with feelings of belonging is collectivistic vs. individualistic orientation [16]. Collectivists can possibly profit more from reminders of their social connections [17]. This is in line with the finding that after reminding participants of Facebook, more collectivistic participants recovered more strongly from social exclusion than did less collectivistic participants [6]. If collectivists profit more from reminders of social connections, they should also cope better with sadness when they are reminded of Facebook.

Building on the findings by Gray et al. [9], we expected that participants would report a greater desire to engage in social activities after seeing a sad (vs. neutral) video. This interest should be diminished after being presented with a Facebook (vs. neutral or positive) icon. The reduction in the desire to engage in social activities after being presented with a Facebook icon should be especially strong for participants high in collectivism. Regarding mood, we expect participants to report less restoration of positive mood after seeing a sad (vs. neutral) video and being presented with a neutral or positive (vs. Facebook) icon. The positive icon condition is included to control for simple positive associations the Facebook icon might evoke. But only the Facebook (vs. positive and neutral) icon should evoke feelings of belonging which can then counteract the heightened desire for social activities and thus restore positive mood to a greater extent. We thus expected a reminder of Facebook to increase feelings of belonging, regardless of the video condition. Within the sad video condition, feelings of belonging should correlate with mood restoration and this should mediate the effect of the icon on mood restoration and the desire to engage in social activities.

## Method

### Participants and design

The study was based on a 2 (Video: sad video vs. neutral video) x 3 (Icon: Facebook vs. Sun vs. Word) between-participants design. Dependent variables were the desire to engage in social activities and mood. Mood was measured twice, once after watching the video and once again after being presented with the icon. Thus, for mood the analyses were based on a 2 (Video: sad video vs. neutral video) x 3 (Icon: Facebook vs. Sun vs. Word) x 2 (Time: Time 1 vs. Time 2) mixed-model design.

We estimated the optimal sample size for the detection of an interaction effect of the IVs based on an effect size of η_p_^2^ = .07 (as in [15]), a power of .80, and an α of .05 with G*Power 3.1 [18]. This resulted in an optimal sample size of *N* = 132.

This study was approved by the ethics committee of the psychology department of the University of Münster. All participants gave informed consent by clicking on a button that stated that they had read the information on the study. In a first round, we collected data from 160 British and U.S. participants via CrowdFlower to allow for sample shrinkage of 20% due to depressive symptoms. Apart from a British or U.S. citizenship, there were no further demographic requirements to participate.

At the beginning of the experiment, participants answered a screening for depressive symptoms. If they showed any depressive symptoms the study was terminated for them after the screening because we did not want to trigger stronger depressive symptoms for these participants in the sad video condition. Based on our threshold for indications of depressive symptoms, 121 out of 160 participants were excluded. We therefore collected data from an additional 480 participants who were again screened for depressive symptoms at the beginning of the study. Of these additional participants, 254 were excluded because they surpassed the threshold of depressive symptoms. Thus, out of all 640 participants, 375 participants were excluded due to depressive symptoms. We discuss this unexpected proportion below. In addition, 128 participants did not complete the study, and two participants were additionally excluded because they had no Facebook account and were in the Facebook icon condition. The final sample size therefore consisted of *N* = 135 participants (74 female, 60 male; mean age = 37.78, *SD* = 13.17). Participants were recruited via CrowdFlower and received $0.50 for participation.

### Procedure and materials

After giving informed consent, participants first stated their demographic details (age, gender, and nationality). The screening for depressive symptoms, which followed immediately afterwards, consisted of two parts: First, participants were asked “During the past month, have you often been bothered by feeling down, depressed, or hopeless?” and “During the past month, have you often been bothered by little interest or pleasure in doing things?” (PHQ-2; [19]; cf. [9]). If participants answered “yes” to one of these questions they were automatically sent to a debriefing page and thus could not participate in the study. Based on this initial screening, 325 participants (51%) were excluded.

Afterwards, the Kessler Psychological Distress Scale [20] was administered to exclude participants who exhibit less specific depressive symptoms but still might be negatively affected by our sadness manipulation. This instrument includes 10 questions about unspecific distress, depression, and anxiety. Specifically, participants are asked to report how often they have experienced various symptoms during the past 30 days (on a scale from 1 = “not once” to 5 = “all of the time”). The rating scores obtained on these ten questions were added up. Scores between 10 and 20 indicate a low risk for depression or an anxiety disorder [21]. Participants with a score greater than 20 were automatically redirected to the debriefing page and excluded from participation in the study. An additional 50 participants (8% of the full initial sample) were excluded with this procedure.

The remaining participants were then randomly assigned to the video condition (sad vs. control video) by the experimental software. They were told to watch the video attentively. In the sad video condition, participants saw a scene from *The Champ*, in which a young boy learns that his father has died. This clip was selected because it has been shown to induce sad mood and increase the desire to engage in social activities [9]. In the neutral video condition, participants saw a trailer for the movie *Island of Lemurs*: *Madagascar*, in which lemurs are portrayed which was chosen because a video about animals has also been used as a control condition in previous research [9] and because it was of similar length (*The Champ*: 2:51 minutes, *Island of Lemurs*: *Madagascar*: 2:31 minutes). After participants had watched the video, we measured general mood with two items (“Are you in a good or bad mood right now” and “How is your mood at the moment?”), rated from 1 (“very bad”) to 7 (“very good”) for the first time (Time 1). These items were highly correlated (*r* = .93, *p* < .001) and integrated into a single score.

Participants then answered the Individualism and Collectivism Scale (INDCOL; [22]) which measures individualism and collectivism with 16 items, respectively. While participants answered this questionnaire the icon manipulation took place. We presented either a Facebook, Word (control neutral) or Sun (control positive) icon in the left corner of the computer screen. Afterwards, the measurement of the dependent variables, that is desire to engage in social activities and mood (Time 2), took place.

To measure the desire to engage in social activities, participants filled out a modified version of the Twenty Statements Test [9]. Participants were asked “to list all the things you would like to do right now”. Therefore, we presented participants with 20 open-ended lines. Two research assistants who were blind to the conditions and hypotheses of the study rated these items regarding whether they were social activities or not. Social activities were defined as either being social by definition (e.g. go on a date) or mentioning another person explicitly or implicitly (e.g., be with my family; cf. [9]). We summed the social activities both for all 20 items and for the first five items [9, 23]. Because only 72 percent of participants listed a total of twenty items, we also calculated percentage scores to control for the amount of listed items. The average number of listed items was 16.28 (*SD* = 6.09). Interrater reliability was high for all measures (*r*s > .90, *p*s < .001).

Mood was again measured with the two items on general mood (*r* = .73, *p* < .001, Time 2) and with the Positive and Negative Affect Schedule (PANAS; [24]). The desire to engage in social activities, general mood, and positive and negative affect were assessed in random order. We then measured participants’ perceptions of general belonging with five items, for example “I do not feel alone in my life” [25] (Cronbach’s α = .77).

Afterwards, participants stated whether they were Facebook users and if so, how many friends they have on Facebook and how much time they spend on Facebook daily. We then asked participants about their relational Facebook use with four items [15] (Cronbach’s α = .84). Subsequently, participants rated their evaluation of Facebook with the items “How important is it to you to have contact via Facebook?”, rated from 1 = “not at all” to 7 = “very important”, and “How do you evaluate Facebook?”, rated from 1 = “very negative” to 7 = “very positive”. The correlation of the two items was sufficiently high to integrate them into a single measure (*r* = .62, *p* < .001). Reminding participants in *all* conditions of Facebook at the beginning might interfere with or preclude potential effects of our icon manipulation. Therefore, the questions about Facebook use were presented at the *end* of the study.

Participants then stated whether they had noticed an icon while answering the questionnaires and if so, which icon they had noticed (no icon, Google, Facebook, Word, Sun, Flash Player, WhatsApp or Skype). Finally, participants answered a suspicion check and were debriefed and compensated.

## Results

### Effects of video-based mood induction and icon presentation on mood and affect

**Fig 1:**
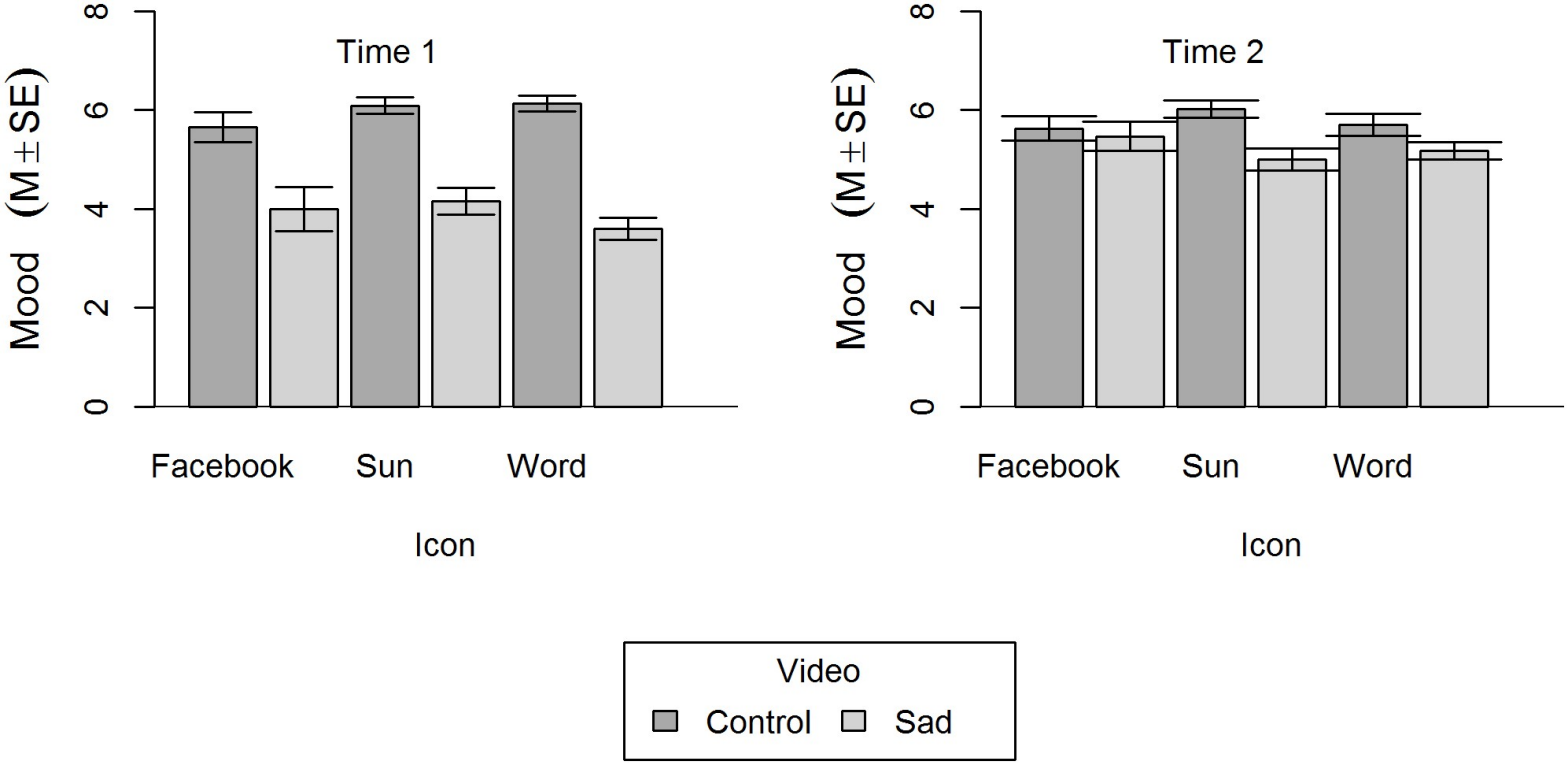
General mood as a function of icon, video, and time. Higher values indicate more positive mood. Error bars depict standard errors.

The effects on general mood were examined with a 2 (Video: sad vs. control) x 3 (Icon: Facebook vs. Word vs. Sun) x 2 (Time: Time 1 vs. Time 2) mixed-model design ANOVA (see Fig 1 for means and *SE*s). There was a significant main effect of Video, *F*(1,129) = 75.44, *p* < .001, η_p_^2^ = .369, as well as a main effect of Time, *F*(1,129) = 20.17, *p* < .001, η_p_^2^ = .135. There was also a significant interaction effect of Time and Video, *F*(1,129) = 48.47, *p* < .001, η_p_^2^ = .273. The analysis yielded no main effect of icon presentation and no interaction effects with icon presentation, *F*s < 2.38, *p*s > .097.

Positive and negative affect from the PANAS scale were both analyzed with a 2 (Video: sad vs. control) x 3 (Icon: Facebook vs. Word vs. Sun) between-participants ANOVAs. For positive mood, there was only a main effect of the presented icon, *F*(2,129) = 4.91, *p* = .009, η_p_^2^ = .071. Participants in the Sun condition (*M* = 2.65, *SD* = 0.87) reported less positive mood than did participants in the Facebook condition (*M* = 3.18, *SD* = 0.84), *t*(80) = 2.75, *p* = .008, *d* = 0.618, and participants in the Word condition (*M* = 3.14, *SD* = 0.91), *t*(99) = 2.72, *p* = .008, *d* = 0.550. Participants in the Facebook and Word condition did not differ in their positive mood, *t*(85) = 0.23, *p* = .821. There was no effect of the presented video and no interaction effect of the two variables on positive mood, *F*s < 1, *p*s > .690. Regarding negative mood, there were no main or interaction effects, Fs < 2.11, ps > .125.

### Effects of video-based mood induction and icon presentation on the desire to engage in social activities

The effects on the desire to engage in social activities were examined with a 2 (Video: sad vs. control) x 3 (Icon: Facebook vs. Word vs. Sun) between-participants ANOVA. There were no main or interaction effects regarding the total number of listed social activities, *F*s < 2.50, *p*s > .119, or the number of social activities among the first five listed activities, *F*s < 2.10, *p*s > .150. Controlling for the number of listed items did not change the results, *F*s < 2.11, *p*s > .14.

To examine whether the sad video increases the desire to engage in social activities [9], we conducted one-tailed t-tests for the total number of social activities and for the number of social activities among the first five listed activities. In total, participants listed marginally more social activities after seeing the sad video (*M* = 2.38, *SD* = 2.00) than after seeing the control video (*M* = 1.86, *SD* = 1.70), *t*(132) = 1.62, *p* = .054, *d* = 0.283. Among the first five activities, participants also listed marginally more social activities in the sad video condition (*M* = 0.69, *SD* = 0.97) than in the control video condition (*M* = 0.49, *SD* = 0.75), *t*(132) = 1.35, *p* = .091, *d* = 0.234.

### Moderation by Facebook use and by individualism and collectivism

We examined whether the effects of the experimental manipulations on general mood and the desire to engage in social activities were moderated by time spent on Facebook, number of Facebook friends, evaluation of Facebook, relational Facebook use, as well as individualism, and collectivism. Because the video-based mood manipulation had no effect on positive and negative affect, these data were not further examined. We applied the Bonferroni adjustment of the significance threshold to correct for possible Type I error inflation (false positive test results) due to testing of multiple moderators. Specifically, for our tests of the six potential moderator variables, we considered *p*-values lower than .008 as significant and *p*-values smaller than .017 as marginally significant.

There was one extreme outlier for time spent on Facebook (eight hours/day) and for the number of Facebook friends (4050 friends). To avoid the disproportional influence of this outlier, the data from this participant were eliminated for the analyses regarding time spent on Facebook and number of Facebook friends.

For general mood, a difference score was calculated (Mood Time 2 –Mood Time 1) for an estimate of mood restoration. There were no moderation effects by time spent on Facebook, number of Facebook friends, evaluation of Facebook, and relational Facebook use, *t*s < 1.65, *p*s > .100. There were also no moderation effects of any of the four Facebook use variables on the desire to engage in social activities, *t*s < 1.66, *p*s > .100.

**Fig 2:**
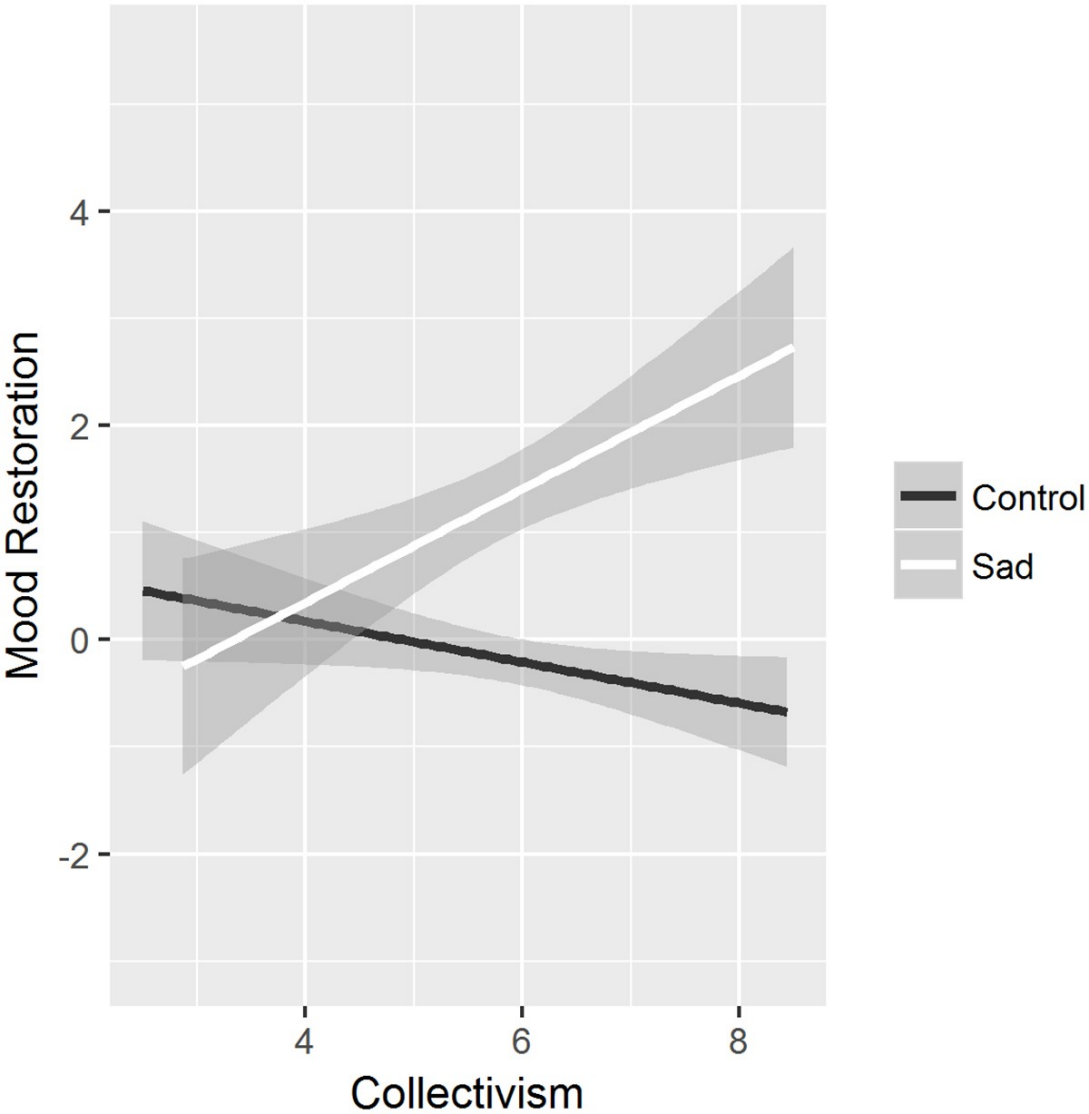
Restoration of general mood as a function of video (control vs. sad) and collectivism. Grey areas represent 95% CIs, dark grey areas represent overlapping CIs.

We then tested possible moderation effects of collectivism and individualism. Regarding the desire to engage in social activities, there were no moderation effects of collectivism and individualism, *t*s < 1.14, *p*s > .260. Regarding general mood restoration (i.e., the mood difference score), there was no moderation effect by individualism, *t*s < 1.44, *p*s > .152. There was, however, a significant two-way interaction between video and collectivism, *b* = 1.04, *t*(123) = 3.04, *p* = .003. There was a significant main effect of the video for participants high in collectivism (*M* + 1 *SD*), *b* = 2.67, *t*(123) = 4.81, *p* < .001, but not for participants low in collectivism (*M*—1 *SD*), *t*(123) = 0.44, *p* = .665 (Fig 2). Thus, higher collectivistic orientation was associated with greater restoration of positive mood after watching a sad movie. There were no main or interaction effects of the icon, *t*s < 1.76, *p*s > .082.

## Effects of video-based mood induction and icon presentation on feelings of belonging

To examine whether a Facebook reminder affects participants’ feelings of belonging, we conducted a 2 (Video: sad vs. control) x 3 (Icon: Facebook vs. Word vs. Sun) between-participants ANOVA with feelings of belonging as dependent variable. The analysis yielded no main effect of the video, *F*s < 2.22, *p* > .110, and a marginally significant interaction effect between presented icon and video, *F*(2,129) = 2.55, *p* = .082, η_p_^2^ = .038 (Table 1). A closer inspection of this interaction effect produced the following findings: The presented icon had a significant effect on belongingness feelings after participants had watched a sad video, *F*(2,57) = 4.10, *p* = .022, η_p_^2^ = .126. In contrast, the icon manipulation had no effect after participants had watched the control video, *F*(2,72) = 1.41, *p* = .251, η_p_^2^ = .038. Within the sad-video condition, participants reported higher levels of belonging after seeing the Facebook icon compared to the Sun icon, *t*(32) = 2.50, *p* = .018, *d* = 0.817, and after seeing the Word icon compared to the Sun, *t*(45) = -2.32, *p* = .026, *d* = 0.712.

**Table 1:** Means (with standard deviations) for feeling of belonging as a function of icon presentation (Facebook vs. Sun vs. Word) and video (sad vs. control).

	Facebook icon	Sun icon	Word icon
Sad Video	5.51 (0.80)	4.68 (1.14)	5.41 (0.93)
Control Video	5.57 (1.10)	5.26 (1.39)	4.96 (1.18)

We then examined whether the Facebook icon induced greater feelings of belonging than both the Sun and the Word icon. To this end, we conducted one-tailed t-tests between the Facebook and the Sun and Word icon, respectively. Participants in the Facebook condition (*M* = 5.55, S*D* = 0.98) reported significantly more feelings of belonging than participants in the Sun condition (*M* = 5.02, *SD* = 1.31), *t*(81) = 2.10, *p* = .019, *d* = 0.447, and marginally more than participants in the Word condition (*M* = 5.18, *SD* = 1.08), *t*(86) = 1.65, *p* = .051, *d* = 0.355.

Feelings of belonging were unrelated to mood restoration, both in the sad video condition, *r* = .148, *p* = .258, and in the control condition, *r* = .139, *p* = .236. Because there was also no effect of the icon on the desire to engage in social activities, the possible mediation of the effect of the icon on the desire to engage in social activities in the sad video condition was not examined further. Feelings of belonging were positively correlated with the total numbers of listed social activities in the sad video condition, *r* = .242, *p* = . 062. This correlation was significant when controlling for the number of listed activities, *r* = .276, *p* = .033. The correlation was not significant for the first five listed activities, *r* = .173, *p* = .187. In the control condition, feelings of belonging and number of listed social activities (total and among the first five activities) were not correlated, *r*s < .041, *p*s > .736. Because there was no effect of the icon in the sad video condition and belonging was positively correlated to the desire to engage in social activities rather than negatively, as expected, this mediation was also not examined further.

There were no moderation effects by indicators of Facebook use on the feeling of belonging, *t*s < 1.55, *p*s > .120. There were also no moderation effects by collectivism or individualism, *t*s < 1.72, *p*s > .089.

### Additional analyses

Only 48 participants (36%) correctly identified the icon. There were no two- or three-way interaction effects of correct identification of the icon with icon presentation for general mood, *F*s < 2.43, *p*s > .092, for positive mood, *F*s < 2.44, *p*s > .091, negative mood, *F*s < 1, *p*s > .445, and the desire to engage in social activities (both total and among the first five listed items), *F*s < 1.43, *p*s > .245.

## Discussion

The main hypothesis was not supported. Participants who were presented with a Facebook icon (vs. the neutral or positively valenced icon) did not report less desire to engage in social activities after seeing a loss-related, sad video. We could, however, partly replicate findings by Gray [9], who found that participants showed a higher desire to engage in social activities after seeing a sad video. Yet, reminders of Facebook did not moderate this effect.

Participants’ general mood was lower right after seeing the sad video, indicating that the induction of negative mood was successful. However, the restoration of general mood did not differ between the icon conditions. Thus, subtly reminding participants of Facebook did not influence their response to sad mood.

A critic might argue that the lack of an effect of icon presentation was due to the low proportion of participants who noticed the icon. However, this proportion is quite similar to proportions found in previous studies using the same methodology [6; 15]. In these previous studies, the presentation of a Facebook icon reduced interest in social contact and increased belongingness needs after social exclusion regardless of whether participants noticed (or did not notice) the Facebook icon. In the present study, the presentation of the Facebook icon led to higher belongingness feelings although the majority of participants did not consciously notice the icon. The subtle reminder of Facebook was, thus, sufficient to increase feelings of belonging. This finding is in line with a substantial body of research showing that knowledge can be activated incidentally (e.g., [26; 27]), or even unconsciously [28], by subtle cues in the perceiver’s environment. Recent critiques of so-called priming effects notwithstanding, recent research confirms that incidentally or automatically activated knowledge can affect the perceiver’s cognition and behaviors (see [29; 30]).

Of course, it is possible that some of our participants noticed the icon but did not consciously remember it later in the study session. Future research could use eye tracking to assess the extent to which participants look at the icon during presentation.

Although the majority of participants could not consciously remember the icon, the icon presentation still affected feelings of belonging. As predicted, participants reported higher feelings of belonging after seeing a Facebook (vs. Word or positive) icon. Thus, this study is in line with previous findings that subtle Facebook reminders can increase feelings of belonging after social exclusion [6].

Previous research has shown that participants use Facebook as a coping strategy when they are experiencing feelings of disconnectedness [13]. Supporting this notion, we found that subtle reminders of Facebook generally increased feelings of belonging, even without prior activation of loss-related sadness. Thus, reminding people of Facebook may generally enhance people’s sense of connectedness, regardless of whether their belongingness needs have been threatened. This finding testifies to the potency of thoughts about one’s connectedness via social online media. This being said, future research should re-examine the interaction between loss-related sadness and reminders of social online media with alternative manipulations.

Somewhat unexpectedly, we found that feelings of belonging were positively correlated with the desire to engage in social activities. Thus, experiencing a sense of belonging may elicit the motivation to behave accordingly, that is, to seek and enjoy corresponding social activities with their friends. This might explain why we did not find that Facebook diminished participants’ desire to engage in social contact in the sad-video condition. Specifically, Facebook reminders may trigger two processes that have partly incompatible effects. On the one hand, when people suffering from loss-related sadness are reminded of Facebook, they do not need to engage in compensatory social contact [6]. On the other hand, Facebook reminders generally increase people’s feeling of belonging, which in turn enhances their motivation to engage in corresponding social activities.

In our study, the desire to engage in social activities was measured with the Twenty Statements Test. This was chosen as a dependent variable in order to replicate the findings by Gray et al. [9]. However, this dependent variable may be confounded with the activation of thoughts of social connections. If reminding people of Facebook activates thoughts of their social connections on Facebook, they might be more likely to name activities that include these social connections. This might actually not reflect a higher desire to engage in social activities but activities that include their social connections might simply be more salient. Another issue with this dependent variable is that participants on average only listed about two social activities. Thus, it is possible that a floor effect occurred making the detection of effects of the icon presentation less likely. Therefore, in future studies, it would be important to measure the desire to engage in social activities with a measure that is independent of the saliency of social connections and less likely to lead to a floor effect.

A surprising observation in our study was the very high exclusion rate due to depressive symptoms of about 59% of initial participants. This is an extremely high rate, given that the 12-month prevalence rate for depression is about 7% [31]. There are several potential reasons for this high exclusion rate. First of all, we used a rather strict criterion by excluding participants if they affirmatively answered one of the questions of the PHQ-2 [19]. The same exclusion criterion has been used before [9], and was also found to lead to a high exclusion rate of 42% in a student sample. Additionally, we excluded participants if they reported a score higher than 20 on the Kessler Psychological Distress Scale [20], which led to the exclusion of an additional 8% of participants. This number is quite similar to other findings of the prevalence of these symptoms in the general population [21]. However, note that many participants who had already been excluded on the basis of the first screening (51%, screened with the PHQ-2) would probably score higher on the Kessler Psychological Distress Scale.

The high proportion of depressive symptoms in our initial sample is in line with findings from Mechanical Turk studies, which show prevalence rates of depression and anxiety three to nine times *higher* than the 12-month prevalence rate in the general population [32]. We assume that participants on CrowdFlower might have similar high depression rates as participants on Mechanical Turk. Thus, when recruiting participants on these platforms, researchers should be careful about the generalizability of these results and the potential harm that manipulations of emotions could do to their participants. Because we excluded all participants who exhibited any symptoms of depression, an overrepresentation of participants with depressive symptoms is not an issue.

Conversely, it is possible that participants with more depressive symptoms might have responded more strongly to the sad video and that they might have been affected differently by the reminders of Facebook. Future research should examine this possibility.

We expected participants scoring high on collectivism to profit from the Facebook icon to a greater extent [6]. However, because Facebook reminders did not influence participants’ response to the sad video, it is not surprising that collectivism did not influence the results. The only exception we found is that collectivists generally recover more strongly from the sad mood induction, such that they show a higher recovery of positive mood. To our knowledge, this is the first time that this relationship was found. Previous studies found that people from collectivistic cultures generally express less emotions [33] and report less emotional intensity of sadness [34]. Our finding goes beyond this because we did not observe differences in the initial emotional reaction but differences in the restoration of positive mood.

The sadness induced in our study was related to a social loss [9]. To further understand the relationship between collectivism and the restoration of positive mood it would be interesting to examine whether this effect only occurs for loss-related sadness. It is possible that more collectivistic participants can generally recover more quickly from a sad mood regardless of whether the sad mood is socially motivated. In other words, a stronger (vs. weaker) collectivistic orientation may be associated with capacities or personality characteristics that allow people to overcome sad moods, such as wellbeing or openness to new experiences. However, it seems more plausible that the activation of social connections facilitates coping with sadness for more (vs. less) collectivistic participants. As collectivists may profit more from reminders of social connections [6, 17], they might therefore recover better from sad mood induction.

By this logic, reminders of social connections should help people cope with a sad mood. However, in the present study reminders of the social network Facebook had no impact on mood recovery. This might be due to the relative subtlety of the Facebook icon manipulation: Compared to the possible activation of social connections induced by the video about social loss, the marginal presentation of the Facebook icon was possibly too subtle to influence participants’ coping with sadness. Thus, it may be possible that stronger reminders of social connections are necessary to attenuate the need for social reconnection. Therefore, in future studies it should be examined whether actively thinking about social connections or active Facebook use can counteract effects of sadness.

To conclude, we found that a reminder of Facebook increased participants’ feelings of belonging. We could also replicate that sad mood induction increases the desire to engage in social activities. However, responses to sadness were not influenced by reminding participants of Facebook. Possibly, stronger reminders of social connections might be necessary to enhance coping with sadness.
